# Fusion of FDG-PET Image and Clinical Features for Prediction of Lung Metastasis in Soft Tissue Sarcomas

**DOI:** 10.1155/2020/8153295

**Published:** 2020-05-05

**Authors:** Jin Deng, Weiming Zeng, Yuhu Shi, Wei Kong, Shunjie Guo

**Affiliations:** College of Information Engineering, Shanghai Maritime University, 1550 Haigang Ave., Shanghai 201306, China

## Abstract

Extracting massive features from images to quantify tumors provides a new insight to solve the problem that tumor heterogeneity is difficult to assess quantitatively. However, quantification of tumors by single-mode methods often has defects such as difficulty in features extraction and high computational complexity. The multimodal approach has shown effective application prospects in solving these problems. In this paper, we propose a feature fusion method based on positron emission tomography (PET) images and clinical information, which is used to obtain features for lung metastasis prediction of soft tissue sarcomas (STSs). Random forest method was adopted to select effective features by eliminating irrelevant or redundant features, and then they were used for the prediction of the lung metastasis combined with back propagation (BP) neural network. The results show that the prediction ability of the proposed model using fusion features is better than that of the model using an image or clinical feature alone. Furthermore, a good performance can be obtained using 3 standard uptake value (SUV) features of PET image and 7 clinical features, and its average accuracy, sensitivity, and specificity on all the sets can reach 92%, 91%, and 92%, respectively. Therefore, the fusing features have the potential to predict lung metastasis for STSs.

## 1. Introduction

Sarcomas are a highly heterogeneous group of tumors classified according to the similar adult tissue types in tissue occurrence [1]. It is characterized by invasive or destructive growth that can recur and by distant metastasis [2]. As one of the sarcomas, soft tissue sarcomas (STSs) can occur anywhere in the body, and 59% of which originate in an extremity [3]. Unfortunately, 10%-20% of the patients with sarcomas or STSs have distant metastasis at the time of diagnosis. The metastasis rate is approximately 30%-40% in the course of follow-up, of which lung metastasis accounts for about 90% [4–6]. Moreover, there is a great deficiency in the cognition of the prognostic factors of lung metastatic tumor resection and the recurrence rate after resection is high [7]. Therefore, early screening and prediction of lung metastasis can help patients with STSs find corresponding self-treatment measures at an early stage and improve the survival rate of patients.

The most common method to evaluate the risk of lung metastasis is to study the heterogeneity of tumors from histopathological samples, while the biological relationships between different clonal subgroups or clones and microenvironments in solid tumors such as STSs are still unclear, so that the information obtained from the samples is affected by the sampled region, which is not necessarily representative [8]. Therefore, it is hard to study the heterogeneity of tumors from the point of view at the molecular level because solid cancers are spatial and temporal heterogeneous. Lambin et al. have suggested that extracting a large number of features from medical images can solve this problem because the radiomic feature has the ability to capture intratumoural heterogeneity in a noninvasive [9]. Several studies have predicted the effect of lymph node metastasis and adjuvant radiotherapy or chemotherapy in preoperative colorectal cancer by using radiomic features [10, 11]. Corino et al. performed radiomic analysis of STSs to distinguish moderate and high lesions [12]. Valliã¨Res et al. extracted a large number of texture features from 2-deoxy-2-[18F]fluoro-D-glucose (FDG) positron emission tomography (PET) and magnetic resonance imaging (MRI) data for the construction of a STS lung metastasis prediction model [13]. However, the cost of acquiring multiple modal images at the same time is high and may not be affordable for some patients. Also, the image acquisition of different modes is complex, and different image information sets tend to obtain several overlapping feature information. In addition, the acquisition of a large amount of information increases the complexity of the model construction and the time complexity of the operation. The features obtained from only a single image are limited, and more other accessible modal data may be overlooked such as clinical data. Actually, the fusion of image and other modal information can help us to obtain features from multiple aspects that are used to build a more accurate and stable model. There are a few studies that have used the multimodal method to achieve great results; for example, Aerts et al. quantified the tumor microenvironment by fusing imaging, gene, and clinical information to quantify tumor gene heterogeneity in the early stage [14]. Tingdan et al. developed and validated a clinical radiomics nomogram for preoperative prediction of lung metastasis in colorectal cancer patients [15].

PET image is a kind of reaction molecular metabolism imaging. When the disease is changing in the early stage of the molecular level and the morphology of the lesion area has not been abnormal, the lesion can be found by PET examination. Compared with other types of images, PET has the characteristics of high sensitivity, high specificity, and better security [16]. Therefore, this study extracted the features from two kinds of data including PET image and clinical data, and then the feature fusion was performed, which was applied to the subsequent prediction model construction. For the problem of model construction, it is expected that the predictive model can be as simple as possible and has a good predictive effect. The aim of the simple model is actually to expect it to be used as a representative feature as possible for the model construction. Hence, it is particularly important to choose higher importance features from a large number of features. There are two functions for feature selection including reducing the number of features and lowering the dimension of features, which are both used to make the model generalization more powerful by reducing the overfitting and by enhancing the understanding of features. Taking into account that the random forest algorithm has the ability to analyze the features of complex interaction classification and has good robustness for noise data or data with missing values, its variable importance measure can be used as a feature selection tool for high-dimensional data [17]. Therefore, the random forest method is applied to extract higher-contribution features from multimode data. Then, they are used as the input for a back propagation (BP) neural network with the superior ability of nonlinear mapping, self-learning, self-adaptive, generalization, and fault tolerance to construct the lung metastasis prediction model [18]. The results showed that the model constructed by combining the features of image and clinical data has a better performance in all data sets. Furthermore, it could be found that only the top PET features and clinical features achieved a higher accuracy rate of more than 90%. These features are strongly correlated with lung metastasis and may be used as a label for lung metastasis prediction of STSs.

## 2. Methods

### 2.1. Data Sources and Preprocessing

The FDG-PET imaging data of 51 patients with STSs were included in this study, and the corresponding clinical data were downloaded from The Cancer Imaging Archive (TCIA). All patients underwent pretreatment FDG-PET scanned between November 2004 and November 2011, during which 19 patients developed lung metastases [13]. The FDG-PET slice thickness resolution was 3.27 mm for all patients and the median inplane resolution was 5.47 × 5.47 mm^2^ (range: 3.91-5.47 mm). The details of samples could be found in Table 1.

We divided these samples into two groups, *LungMets* and *NoLungMets*. *NoLungMets* were patients that did not develop lung metastases and *LungMets* were patients that eventually developed lung metastases. The FDG-PET imaging data also contained contours of the 3D tumor region of each sample drawn by an expert radiation oncologist. To better understand the difference of images between the two groups, the region of interest (ROI) is a tumor area which is extracted according to the lesion contour mask mapped onto the original image. Therefore, ROI volumes of 51 patients are obtained for further analysis.

### 2.2. Feature Extraction

Considering that the standard uptake values (SUV) as a semiquantitative index in PET can be effectively applied in the evaluation of benign and malignant tumors and evaluation of curative effect, the FDG-PET volume is first converted to SUV maps and then a square root transform is applied to help stabilize the PET noise in the image.

These features are extracted from three aspects, and all of which are derived from the ROI regions. In Table 2, there are 67 features including 5 SUV metrics features, 5 types of texture features, and 6 types of clinical features. The study uses five types of texture features, namely, global, gray-level co-occurrence matrix (GLCM) [19], gray-level run-length matrix (GLRLM) [20–23], gray-level size zone matrix (GLSZM) [20–23], and neighborhood gray-tone difference matrix (NGTDM) [24].

The corresponding feature vector is calculated and obtained for each feature. SUV-related features can be obtained by simple mathematical calculations. Each extraction method of texture feature has a corresponding calculation formula, which can be obtained by corresponding references [19–24]. Then, we calculated all the texture feature values corresponding to each sample by the formula of each texture feature. Furthermore, considering that the most clinical features are presented in text form, a coding method called one-hot is applied to extract text features [25]. For example, the *Sex* feature includes male and female, the male is represented as 1 0 and the female is denoted as 0 1. Similarly, feature *Status* is coded as Alive (1 0 0), Alive with disease (0 1 0) and Dead (0 0 1). These feature vectors make up a feature matrix with a size of 51 × 67. Row denotes the sample and each column is a feature vector.

### 2.3. Feature Selection Based on Random Forest

A random forest is an integrated classifier with a set of decision tree classifiers that can be expressed as {*h*(*X*, *θ*_*k*_), *k* = 1, 2, ⋯, *K*}, where {*θ*_*k*_} is a random vector obeying independent and identical distribution. *K* represents the number of decision trees in random forest. The optimal classification result is determined by the voting of each decision tree classifier when given an argument *X* [17].

The variable importance assessment is a significant feature of random forest algorithms. In this study, we use a variable importance measure based on the classification accuracy of out-of-bag (oob) data. The evaluation criterion of this method is the average reduction of the classification accuracy after the slight disturbance of the independent variable of the oob data and classification accuracy before the disturbance.

Assuming that there are bootstrap samples *b* = 1, 2, ⋯., *B*, where *B* represents the number of training samples, the variable importance$\bar{D_{j}}$of feature *X*_*i*_ based on classification accuracy is calculated by the following steps: Firstly, the decision tree *T*_*b*_ is constructed based on training samples after setting the value of *b* to 1, and then the oob data is defined as *L*_*b*_^*oob*^. After that, the oob data is classified by using the *T*_*b*_, and the number of correct classifications is calculated as *R*_*b*_^*oob*^. For feature *X*_*j*_(*j* = 1, 2, ⋯, *N*), the value of the feature *X*_*j*_ in *L*_*b*_^*oob*^is disturbed, and the data set after disturbance is defined as *L*_*bj*_^*oob*^. The number of *L*_*b*_^*oob*^is classified by *T*_*b*_, and the count of correct classifications is calculated as *R*_*bj*_^*oob*^. The same steps are performed on other features. Finally, the importance $\bar{D_{j}}$of feature *X*_*j*_ is calculated by the formula

(1) $$\begin{array}{c} \bar{D_{j}}=\frac{1}{B}\overset{B}{\underset{i=1}{\sum }}\left(R_{b}^{oob}-R_{bj}^{oob}\right). \end{array}$$

### 2.4. Back Propagation (BP) Neural Network Model

The principle of BP neural network is that the gradient descent method is used to adjust the weights and thresholds, so that the mean square error value of the actual output of the network and the expected output is minimal. The training simulation process is presented as follows.

Firstly, the BP neural network structure is determined and the input layer to the implicit layer weight value *w*_*ij*_, the implicit value to the output layer weight *v*_*ij*_, the implicit layer threshold *θ*_*j*_, and the output layer threshold *ɤ*_*t*_ are assigned. Then the training samples (*P*^*k*^, *R*^*k*^) are randomly selected to provide to the network. After that, the input of each element of the implicit layer *S*_*j*_ is calculated by using the output sample *P*^*k*^, the connection weight value *w*_*ij*_, and the threshold value *θ*_*j*_, and then the output *B*_*j*_ of the implicit layer unit is calculated by using the *S*_*j*_ through the transfer function as follows:

(2) $$\begin{array}{c} S_{j}=\overset{m}{\underset{i=1}{\sum }}w_{ij}p_{i}^{k}-\theta_{j},\\ B_{j}=f\left(S_{j}\right). \end{array}$$

Next, the output *L*_*t*_ of the output layer units is calculated using the output *B*_*j*_, weight value *v*_*jt*_, and threshold *ɤ*_*t*_ of the middle layer, and then the response *C*_*t*_ of the output layer unit is calculated by the transfer function so that the following formulas are obtained:

(3) $$\begin{array}{c} Lt=\overset{n}{\underset{j=1}{\sum }}v_{jt}B_{j}-\gamma_{t},\\ C_{t}=f\left(L_{t}\right). \end{array}$$

The generalization error *d_t_* of the output layer can be calculated using the expected output *R*^*k*^ and the network actual output *C*_*t*_, and the generalization error *e*_*j*_ of each unit in the middle layer can be also calculated based on three parameters including *v*_*jt*_, *d*_*t*_, and *B*_*j*_.

(4) $$\begin{array}{c} d_{t}=\left(r-C_{t}\right)\cdot C_{t}\left(1-C_{t}\right)\\ e_{j}=\left[\overset{p}{\underset{t=1}{\sum }}d_{t}\cdot v_{jt}\right]B_{j}\left(1-B_{j}\right) \end{array}$$

Then, the connection weight *v_jt_* and threshold *γ*_*t*_ are corrected by using the generalization error *d_t_* of the output layer units and the output *B_j_* of each unit in the middle layer.

(5) $$\begin{array}{c} v_{jt}\left(N+1\right)=vjt\left(N\right)+\alpha \cdot d_{t}\cdot B_{j},\\ \gamma_{t}\left(N+1\right)=\gamma_{t}\left(N\right)+\alpha \cdot d_{t}. \end{array}$$

Therefore, the fixed connection weight *w*_*ij*_ and threshold *θ*_*j*_ can be obtained as follows:

(6) $$\begin{array}{c} w_{ij}\left(N+1\right)=w_{ij}\left(N\right)+\beta \cdot e_{j}\cdot p_{i},\\ \theta_{j}\left(N+1\right)=\theta_{j}\left(N\right)+\beta \cdot e_{j},0<\beta <1. \end{array}$$

Furthermore, the next train sample is randomly selected to provide to the network according to the previous method of training until the training samples are fully trained.

## 3. Results

### 3.1. Feature Selection Based on Random Forest

**Figure 1:**
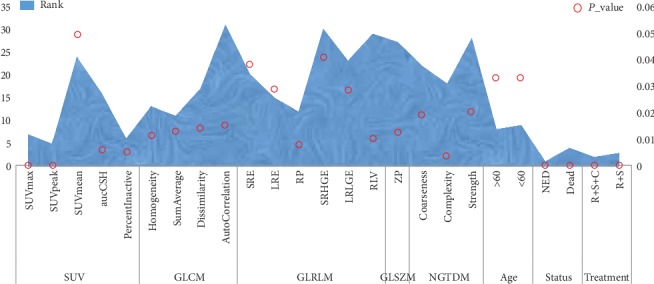
The 25 significant features based on random forest and *T* test. The transverse axis represents different characteristics, the main longitudinal axis represents the ranking of the feature, and the secondary longitudinal axis represents the significance level of the feature.

Feature selection can not only improve the performance of the model but also help us to understand the characteristics of the data and the underlying structure, which plays a significant role in the further improvement of model and algorithm. In this study, the number of trees in random forest is set to 250, and then there are 67 features including 5 SUV metrics features, 5 types of texture features, and 6 types of clinical features. In order to explore the contribution of these features to the prediction model, random forest was applied to sort these features. 36 features were selected whose importance values were more than 0.01. Moreover, a *T* test is used to verify the performance of random forest method in this study. 25 significant features were retained in these features by using the *T* test with a confidence level of 95% which are shown in Figure 1.

As shown in Figure 1, these selected features contain 6 clinical features and 19 image features including 5 SUV features and 14 types of texture features. Furthermore, 6 of the top 10 features are clinical features, including age, status, treatment, and MSKCC type, and the other features belong to image features namely SUV features, including SUVpeak, SUVmax, aucCSH, and PercentInactive. Therefore, there is no any texture feature.

### 3.2. BP Neural Network

**Figure 2:**
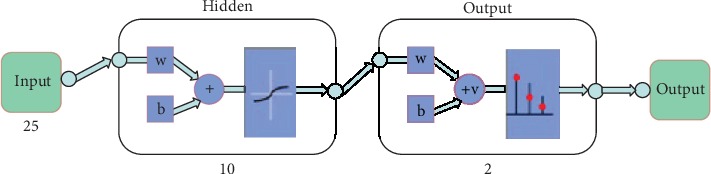
Neural network model. The numbers in the figure represent the number of neurons in each layer.

After obtaining those features, the BP neural network model was constructed, including 1 input layer, 1 hidden layer, and 1 output layer as shown in Figure 2.

For this neural network model, there are 25 most important features so that the number of neurons for input layer is 25. In order to make the model as simple as possible and the time complexity lower, the model in this paper is a simple three-layer model that is the hidden layer is single. The number of neurons for the output layer is 2 because the output layer contains two groups: *LungMets* and *NoLungMets*.

In this study, the *sigmod* function was applied to be the activation function and the method of adaptive learning rate adjustment used to avoid local optimization and overfitting [26]. Then, 51 samples were randomly disrupted and divided into three types according to the proportion of 70%,15%, and 15%, which included 35 training samples, 8 test samples, and 8 validation samples. When the number of iterations reached 1000 times or the gradient value is less than 0.001, the training model was considered to have been trained. Furthermore, in order to overcome the impact of small sample volume and sample specificity on the model, the samples were randomly divided 10 times and then repeated the above process in our study.

**Figure 3:**
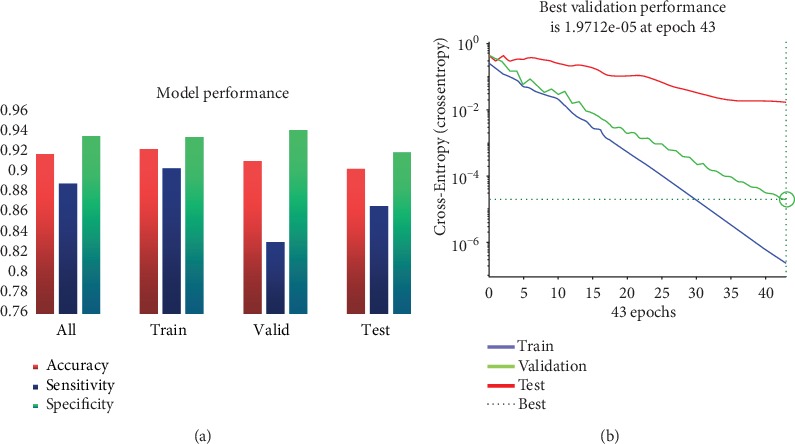
(a) The performance of model including accuracy, sensitivity, and specificity from different data sets. (b) Best validation performance of the neural network.

In order to measure the performance of the model, three indicators were used in this study, including accuracy, specificity, and sensitivity, as shown in Figure 3(a). In addition, the best validation performance of a randomly selected model is shown in Figure 3(b).

It is expected that the model has a high specificity and is sensitive on the basis of high accuracy. In other words, we hope that the model will have a better effect on both *LungMets* and *NoLungMets*. In terms of the total performance of the model, the average accuracy is 92%, and the specificity and sensitivity are 89% and 94%, respectively. Moreover, the model can achieve a good performance in training and validation set as expected. In fact, the results of the test set are more concerned because the model construction is based on the training set and the validation set; the test set is actually not involved in the construction of the model and completely independent of other sets. Therefore, the result of the test set is the standard of model performance evaluation; it can be seen from Figure 3(a) that the average accuracy, specificity, and sensitivity of the test set reached 90%, 87%, and 92%, respectively. In addition, the best validation performance is shown in Figure 3(b); the model has been trained after iterating 43 times, and the gradient mean square error is less than 0.001. At the same time, it can be seen on the validation set that the overall trend of the curve is also in the gradient drop. These evidences not only show that this model has good stability but also show that the selected features can predict the lung metastasis in the soft tissue sarcomas.

## 4. Discussion

**Figure 4:**
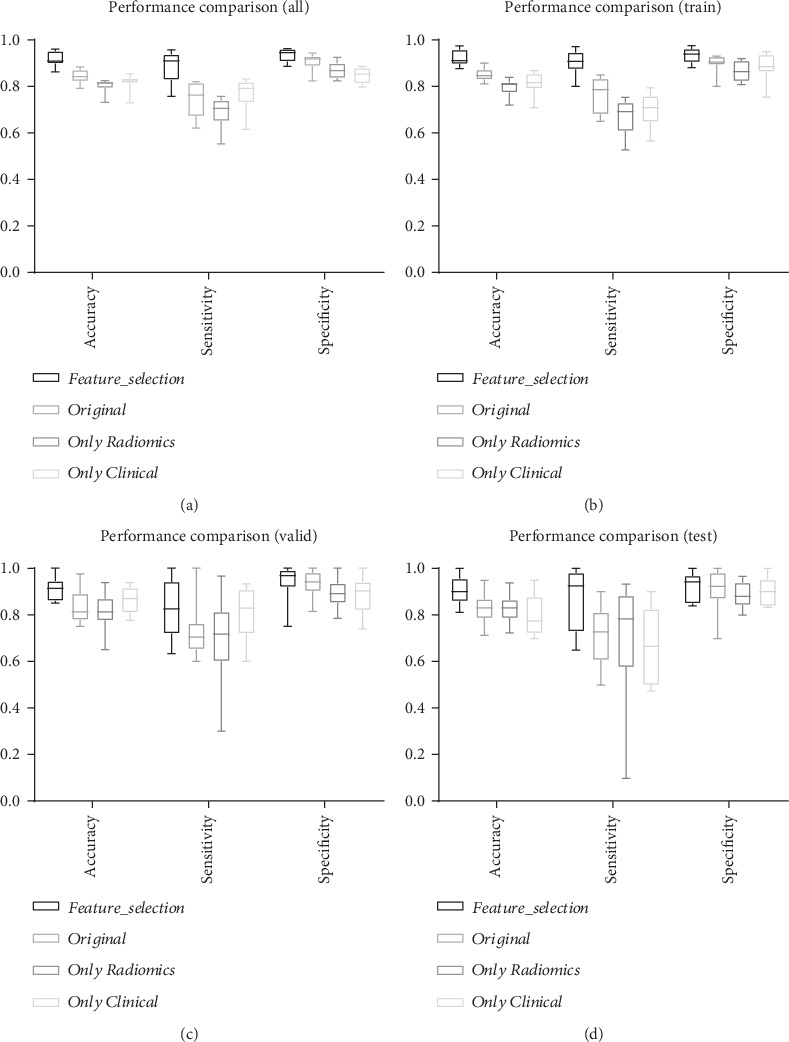
Performance comparison of feature selection. (a) represents the overall performance from the perspective of all data sets. (b–d) denote the performance from the three types of data sets including training, validation, and test set, respectively. *Feature_selection* represents 24 features selected by random forest and *T* test methods. *Original* denotes 67 features including 48 image features and 16 clinical features. *Only Radiomics* represents 48 image features, and *Only Clinical* denotes 16 clinical features.

In order to further confirm whether the prediction effect after feature fusion is really better than that of the single type of features and to avoid the occurrence of chance, this study merely compares the final results of feature fusion with those of image features or clinical features as shown in Figure 4.

Compared with the original features, the effect of prediction is obviously improved based on the selected features by using random forest. For the test set, the prediction accuracy based on the original feature is 83%, while the model prediction accuracy after feature extraction reaches 90%, and the sensitivity of the model is increased by 16%. Therefore, the random forest method can effectively extract the features of higher contribution to the prediction model from the original features and can greatly improve the specificity and sensitivity of the prediction model. In addition, in order to verify the necessity of feature fusion based on multimodal data, the features of single modal data are used to establish a prediction model. The evaluation results of model according to the three measurement indices show that the prediction performance of the feature fusion model is better than that using a single class of modal data at the level of all data sets. For the test set, the multimodal features can obtain higher accuracy and specificity, and although image features also reveal a high average value of sensitivity, it cannot characterize its high sensitivity because the variance is actually too large. Moreover, the sensitivity is not even exceeding 70% in the training and test set because the image features alone are used to construct the model so that the model cannot be trained very well.

**Figure 5:**
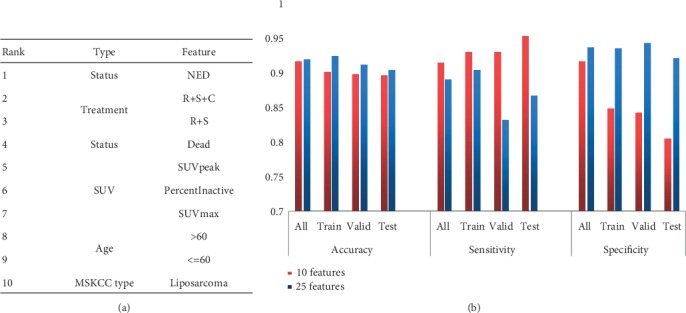
(a) The 10 top features of the contribution degree to prediction model. (b) Comparison of model performance based on different features.

It is worth mentioning that the top 10 features of the 25 features include 3 SUV features and 4 types of clinical features, but these do not contain texture features. Therefore, the prediction model with 10 features as the model input was constructed in this study, and it was compared with the previous model with 25 features as shown in Figure 5.

It can be seen from Figure 5(a) that the top ten features of the contribution ranking are mainly 5 types, of which the image features include three SUV features and the clinical features include the *Status*, *Treatment*, *Age*, and *MSKCC type*. In Figure 5(b), it can be found that the prediction accuracy of the model without texture features decreased by less than 1% compared to the model with 25 features, and the sensitivity increased significantly although the specificity does not seem to be as ideal. These results suggest that the effect of the 10 features is similar to that of the 26 features, which means that it may be not necessary to do a lot of complex texture feature calculations to obtain the same good prediction effect, while the basic clinical features and SUV features are easier to obtain to get than texture features.

**Figure 6:**
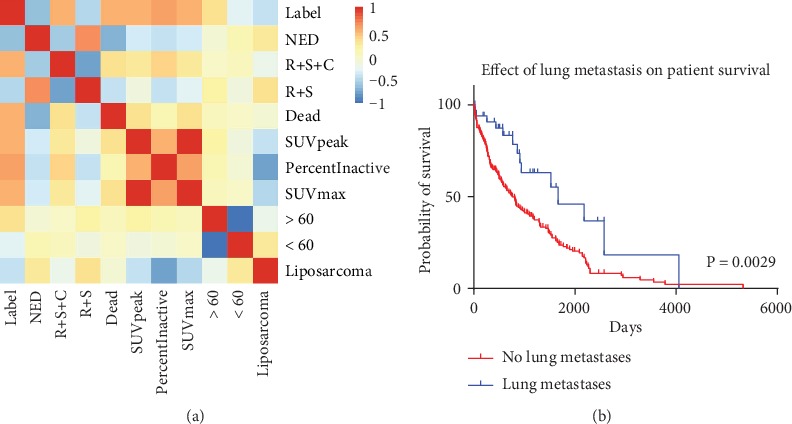
(a) Correlation between features and label. (b) Effect of lung metastasis on patient survival. The blue and red lines represent the survival probability of patients with *LungMets* and *NoLungMets*, respectively. The *x*-axis shows survival time of the patients, and the *y*-axis shows the survival probability of patients.

For PET images, SUV is widely used in the identification and prognosis prediction of benign and malignant tumors. *SUVmax* is always used as the initial index of benign and malignant tumor due to the characteristics of simple operation, good repeatability and not affected by the sketch area of interest. Compared to *SUVmax*, *SUVpeak* overcomes the problem of insensitive to image noise but is sensitive to regions of interest, and *PercentInactive* denotes the percentage of the inactive tumour region. A threshold of 0.005 × (*SUVmax*)^2^ followed by closing and opening morphological operations is used to differentiate active and inactive regions on FDG-PET scans. For patients with lung metastasis, a vast majority of tumors are at a low differentiation stage, and these characteristics of SUV are indicators to distinguish the low differentiation of tumors. Furthermore, we calculated the correlation between these top features and lung metastasis events, which is the label shown in Figure 6(a). In order to verify the relationship between the most contributing feature *Status* and lung metastasis, the clinical data of 254 sarcoma samples with complete information was downloaded from the TCGA database, including 220 samples of *NoLungMets* and 34 *LungMets*, and then these sets of information were applied to calculate the survival curve of sarcoma patients, as shown in Figure 6(b).

It can be seen from Figure 6(a) that there is over medium even strong correlation between most features and label, especially for the *Status*, *Treatment*, and *SUV* features. Moreover, the correlation between features is weak, except for the features of the same types that are very strong, such as *SUVmax, SUVpeak*, *Age*, and *Status*. It is also completely understandable, such as the feature *Age* is either greater than 60 or less than 60. Therefore, these types of nonrelated features are highly representative and can be used as a feature of the prediction model effectively. Figure 6(b) shows that the data of *LungMets* and *NoLungMets* has a significant difference in patient survival time, which demonstrates that the correlation between survival state and lung metastasis is strong. In other words, our results suggest that the feature *Status* is very helpful for the prediction of lung metastasis, and it is easy to obtain the information about the state of survival in practical clinical.

With the development of comprehensive treatment of tumors and the prolongation of survival of cancer patients, the incidence of lung metastatic tumors is increasing. However, in the past, there were few studies on lung metastasis prediction of soft tissue tumors. Valliã¨Res et al. used PET and MRI image data to construct different prediction models, and obtained a considerable prediction accuracy rate by selecting the optimal model [13]. Their study mainly used a large number of texture features with a large number of different parameters. There were more than 9000 texture features extracted from PET data according to different parameters that resulted in excessive time complexity. In our study, the texture features under the optimal parameters were selected, which were merged with SUV features to construct the prediction model for the lung metastasis prediction as shown in Figure 5. It can be drawn from the figure that the performance of models constructed with only image features is significantly lower than that of images and clinical fusion features. Moreover, compared with the best model proposed in [13] based on PET data, the specificity of model was significantly improved, which overcomes the problem of using a large number of texture features. In fact, each texture feature corresponds to a texture feature algorithm, which was complex in the implementation of feature acquisition.

## 5. Conclusion

Based on the complementarity between different modal data features, extracting features from images and clinical data separately provides a new idea to construct predictive models. In this study, the texture and SUV features were extracted from the PET image, and the features of age, gender, and others were extracted from the clinical data. Then, all features were sorted by random forest and two-sample *T* test. The selected features were constructed using the BP neural network to predict the model. The results showed that the performance of multimodal feature fusion was better than that of the image data or clinical data alone. At the same time, the study further analyzed the top 10 significant features, and these features were applied to construct predictive model. It was found that the performance of the model could still achieve the previous effects without the presence of texture features, which were hard to obtain. Furthermore, the method proposed in this study could effectively select high-performance features to construct a prediction model of lung metastasis in STSs, and a high predictive performance was achieved in all data sets. In the future, we hope that this method can integrate more modal data to construct a more effective model to achieve better results, including molecular data such as genes and proteins. At the same time, this method can be extended to other prediction problems such as tumor staging and degree of tumor differentiation.

## Figures and Tables

**Table 1:** Patient and tumor characteristics.

Clinical parameters	
Age, years (mean ± SD)	54.8 ± 16.0
Gender, *n* (%)	
Male	24 (47.1)
Female	27 (52.9)
Histology, *n* (%)	
Malignant fibrous histiocytomas	17 (33.3)
Liposarcoma	11 (21.6)
Leiomyosarcoma	10 (19.6)
Synovial sarcoma	5 (9.8)
Extraskeletal bone sarcoma	4 (7.8)
Fibrosarcoma	1 (2.0)
Other	3 (5.9)
Grade, *n* (%)	
High	28 (54.9)
Intermediate	15 (29.4)
Low	5 (9.8)
Unknown	3 (5.9)
Metastases, *n* (%)	
Lung	19 (37.3)
Other	5 (9.8)
None	27 (52.9)
Time, days (mean ± SD)	
Diagnosis to outcome	285.7 ± 252.3
Diagnosis to last follow-up	849 ± 447.4
Status, *n* (%)	
No evidence of disease	26 (51.0)
Alive with disease	9 (17.6)
Dead	15 (29.4)

Note: SD: standard deviation; *n*: number; diagnosis to outcome: days elapsed between the date of diagnosis of primary STS (biopsy) and the date of diagnosis of recurrence or metastases; diagnosis to last follow-up: days elapsed between the date of diagnosis of primary STS (biopsy) and the date of last-follow-up (or death, if applicable).

**Table 2:** SUV metrics features and Clinical features used in this study.

Type	Name	Description
SUV metrics	SUVmax	Maximum SUV of the tumour region
	SUVpeak	Average of the voxel with maximum SUV within the tumour region and its 26 connected neighbors
	SUVmean	Average SUV value of the tumour region
	aucCSH	Area under the curve of the cumulative SUV volume histogram describing the percentage of total tumour volume above a percentage threshold of maximum SUV
	PercentInactive	Percentage of the tumour region that is inactive. A threshold of 0.005 × (SUVmax)^2^ followed by closing and opening morphological operations were used to differentiate active and inactive regions on FDG-PET scans

Textures	Global	Variance
		Skewness
		Kurtosis
	GLCM	Energy
		Contrast
		Entropy
		Homogeneity
		Correlation
		SumAverage
		Variance
		Dissimilarity
		AutoCorrelation

Textures	GLRLM	SRE (short run emphasis)
		LRE (long run emphasis)
		GLN (gray-level nonuniformity)
		RLN (run-length nonuniformity)
		RP (run percentage)
		LGRE (low gray-level run emphasis)
		HGRE (high gray-level run emphasis)
		SRLGE (short run low gray-level emphasis)
		SRHGE (short run high gray-level emphasis)
		LRLGE (long run low gray-level emphasis)
		LRHGE (long run high gray-level emphasis)
		GLV (gray-level variance)
		RLV (run-length variance)
	GLSZM	SZE (small-zone emphasis)
		LZE (large-zone emphasis)
		GLN (gray-level nonuniformity)
		ZSN (zone-size nonuniformity)
		ZP (zone percentage)
		LGZE (low gray-level zone emphasis)
		HGZE (high gray-level zone emphasis)
		SZLGE (small-zone low gray-level emphasis)
		SZHGE (small-zone high gray-level emphasis)
		LZLGE (large-zone low gray-level emphasis)
		LZHGE (large-zone high gray-level emphasis)
		GLV (gray-level variance)
		ZSV (zone-size variance)
	NGTDM	Coarseness
		Contrast
		Busyness
		Complexity
		Strength

Clinical	Age	Age
	Sex	Male
		Female
	Treatment	Radiotherapy + surgery + chemotherapy
		Radiotherapy + surgery
		Surgery + chemotherapy
	Status	Alive
		Alive with disease
		Dead
	Grade	High
		Intermediate
		Low
	MSKCC type	Liposarcoma
		Leiomyosarcoma
		Synovial sarcoma
		Malignant fibrous histiocytomas
		Extraskeletal bone sarcoma
		Fibrosarcoma
		Other

## Data Availability

The data used to support the findings of this study are from previously reported studies and public database, which have been cited.
